# Beyond the Splanchnic Area: Extra-splanchnic Thrombosis in Acute Pancreatitis

**DOI:** 10.7759/cureus.64555

**Published:** 2024-07-15

**Authors:** Varsha Shinde, Pranay Penmetsa, Yash Dixit

**Affiliations:** 1 Emergency Medicine, Dr. D. Y. Patil Medical College, Hospital and Research Centre, Dr. D. Y. Patil Vidyapeeth (Deemed to be University), Pune, IND; 2 Emergency Medicine, Dr. D. Y. Patil Medical College, Hospital and Research Centre, Dr. D. Y. Patil Vidyapeeth (Deemed to be University, Pune, IND

**Keywords:** point-of-care-ultrasound, anticoagulation, extra-splanchnic, thrombosis, acute pancreatitis

## Abstract

Acute pancreatitis (AP) is an inflammatory condition with varied clinical presentations. Local complications include peripancreatic fluid collection, acute necrotic collection, walled-off necrosis, and pancreatic pseudocyst, but vascular complications like pseudoaneurysm and venous thrombosis are also reported. Patients often experience splanchnic venous thrombosis, which can affect the splenic vein, portal vein, and superior mesenteric vein individually or in combination. Rarely, extra-splanchnic venous thrombosis, including renal vein, superior vena cava, and inferior vena cava thrombosis, has been reported in cases of chronic pancreatitis. The formation of a venous thrombus in acute pancreatitis is multifaceted, with pancreatic inflammation and the immune response mounted by the patient playing a significant role. There is a dearth of medical literature regarding extra-splanchnic venous thrombosis and the use of therapeutic anticoagulation in the successful treatment of the above-mentioned complication. This case report highlights the rare complications that can be seen in cases of acute pancreatitis.

## Introduction

Acute pancreatitis (AP) is an inflammatory condition of the pancreas arising from various causes, with alcohol and gallstones being the most common aetiologies [1]. The pathophysiology of acute pancreatitis is complex, involving the premature activation of pancreatic enzymes, which can result in autolysis of the pancreatic tissue and other tissues surrounding it, ultimately triggering a systemic inflammatory response that can ultimately lead to multiple organ failure and death [2].

Acute pancreatitis is classified as mild or severe. The former is defined as an interstitial edoema of the diseased gland with minimal organ dysfunction. The latter is a severe variant of the disease, seen in 5-10% of patients, and is associated with necrosis of the pancreas, a dysregulated host systemic inflammatory response often leading to shock, electrolyte disturbances, abdominal compartment syndrome [3], and further leading to multi-organ failure, disseminated intravascular coagulation, and death [4]. The mortality rate in patients with acute pancreatitis varies depending on its severity. Mild cases have a low mortality rate, often less than 1%, while severe cases can have a mortality rate as high as 30% to 40% [5].

Complications of acute pancreatitis include local complications like pancreatic necrosis, acute necrotic collection, walled-off necrosis, and pseudocyst formation, while systemic complications include organ failure and systemic inflammatory response syndrome (SIRS). One of the rare complications of pancreatitis is vascular thrombosis, which carries high mortality and morbidity. The splanchnic circulation comprises the portal vein, splenic vein, and superior mesenteric vein. These vessels can get thrombosed due to their close proximity to the pancreas. Extra-splanchnic venous thrombosis rarely occurs as a complication of AP. There is paucity in the literature regarding extra-splanchnic vessel involvement in AP. Here, we present a case report on extra-splanchnic vessel thrombosis as a complication of AP.

## Case presentation

A 38-year-old male patient with no prior medical history was brought to the emergency department with a history of unilateral upper limb swelling and bilateral lower limb swelling for three to four days. The patient has also had a history of breathlessness since yesterday. On arrival at the emergency department, the patient had a patent airway, a respiratory rate of 22 cycles per minute, and a room air saturation of 84% recorded on pulse oximetry. The patient was tachycardic with a heart rate of 115 bpm and a blood pressure of 132/88 mmHg recorded on the right arm in a supine position with a normal capillary refill time. The patient was immediately started on oxygen via face mask at 5 L/min, after which the saturation had improved to 99%. The patient had a GCS of 15/15 with normal capillary blood glucose levels. On exposure, there was a unilateral swelling of the upper limb extending from the mid-arm to the phalanges, which was pitting in type. The patient also had bilateral pitting pedal edoema extending from the toes to the mid-thigh with no discharge or sinuses.

The primary adjuncts, which included the ECG, chest X-ray, and blood gas, revealed sinus tachycardia on the ECG and type 1 respiratory failure on the arterial blood gas (ABG) in room air (Table 1). A chest X-ray showed blunting of the left costo-phrenic angle with the collapse of the left lower lobe of the lung, suggestive of pleural effusion.

**Table 1:** ABG of the patient. ABG: arterial blood gas.

	Patient	Reference range
pH	7.35	7.35–7.45
pCO_2_	38 mmHg	35–45 mmHg
pO_2_	66 mmHg	75–100 mmHg
Bicarbonate	22 mEq/L	22–26 mEq/L
Lactate	1.1	<2

**Figure 1:**
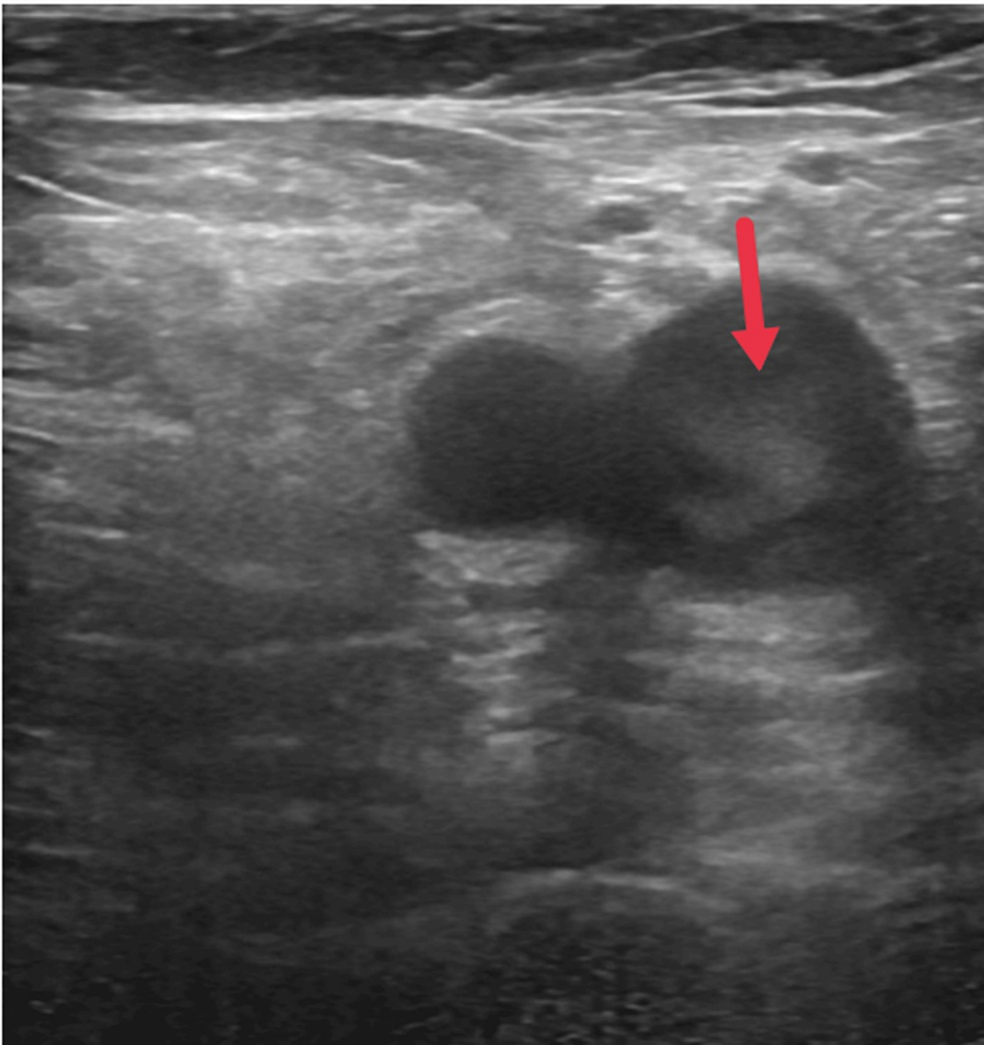
Point-of-care ultrasonography of left lower lower limb showing echogenic thrombus (red arrow) in left femoral vein.

Point-of-care ultrasonography of the lungs showed bilateral lung sliding, with occasional B lines noted in all lung fields, along with a moderate pleural effusion noted in the left lower lobe. Point-of-care ultrasonography and 2D echocardiography reported a normal ejection fraction with no regional wall motion abnormalities. The right atrium and right ventricle appeared dilated, and a tricuspid regurgitation jet was noted with a tricuspid annulus plane systolic excursion (TAPSE) of 12 mm (normal: 18-24 mm), suggestive of right ventricular dysfunction. Point-of-care ultrasonographic screening of the bilateral lower limb revealed a normal study in the right lower limb, but a floating thrombus is noted in the left femoral vein with reduced flow on the colour Doppler (Figure 1).

During the secondary survey, the patient gave a history of unilateral diffuse left upper limb swelling, which was insidious in onset and progressive, started distally in the fingers, and progressed to mid-arm over three to four days, with no discharge, discoloration, or local rise in temperature. The patient also gives a history of bilateral diffuse lower limb swelling extending from the toes to the mid-thigh, which was insidious in onset and progressive with no discharge, sinuses, or local rise of temperature. The patient gives a history of insidious onset breathlessness since 1 day, MMRC grade 1, not associated with seasonal, postural, or diurnal variations.

The patient consumes alcohol daily; the last drink was eight days ago. The patient also gives a history of admission to a prior hospital eight days ago with complaints of severe epigastric pain associated with nausea and vomiting. He was diagnosed with acute pancreatitis, treated conservatively for five days, and discharged home. The patient was alright for one day and then started noticing the above-mentioned symptoms. He had no known allergies and no past medical or surgical history. On examination, the patient had pitting pedal edoema but no pallor, icterus, cyanosis, clubbing, or lymphadenopathy. On respiratory examination, he had reduced air entry in the left lower zone, with normal vesicular breath sounds heard in all other zones and no added sounds. Abdominal examination revealed epigastric tenderness with no radiation. The other system examination was within normal limits. All peripheral pulses were felt equally on both extremities, and no discoloration, sinuses, discharge, or local rise of temperature was noted. Relevant blood investigations were sent, and the results are attached below (Table 2).

**Table 2:** Displaying the bloodwork of the male patient with leukocytosis, deranged amylase, lipase levels and dyslipidemia. aPTT: activated partial thromboplastin time, VLDL: very low density lipoprotein, LDL: low density lipoprotein, HDL: high density lipoprotein, INR: International Normalised Ratio.

Blood workup	Patient	Reference range
Hemoglobin	10.1 g/dl	13.2–16.6 g/dl
Total leukocyte count	27,100/mcl	4000–10,000 mcl
Platelet count	352,000/mcl	150,000–410,000/mcl
Total bilirubin	0.47 mg/dl	0.22–1.2 mg/dl
Conjugated bilirubin	0.28 mg/dl	<0.5 mg/dl
Unconjugated bilirubin	0.19 mg/dl	0.1–1.0 mg/dl
SGOT (AST)	71 U/L	8–48 U/L
SGPT (ALT)	33 U/L	7–55 U/L
Alkaline phosphatase	94 U/L	40–129 U/L
Urea	17 mg/dl	17–49 mg/dl
Creatinine	0.64 mg/dl	0.6–1.35 mg/dl
Sodium	138 mmol/L	136–145 mmol/L
Potassium	4.79 mmol/L	3.5–5.1 mmol/L
Chloride	101 mmol/L	98–107 mmol/L
Calcium	7.8 mg/dl	8.6–10.2 mg/dl
Amylase	457 U/L	25–110 U/L
Lipase	903 U/L	16–77 U/L
Ammonia	108.64 mg/dl	20–100 mg/dl
Prothrombin time	13.5 seconds	10.09–13.79 seconds
aPTT	33.2 seconds	21.76–32.54 seconds
INR	1.13	0.85–1.15
D-dimer	4070 ng/ml	0–500 ng/ml
Homocysteine	7.4 μmol/L	5–15.4 μmol/L
Triglycerides	178 mg/dl	<150 mg/dl
VLDL	36 mg/dl	<30 mg/dl
HDL	24 mg/dl	>40 mg/dl

**Figure 2:**
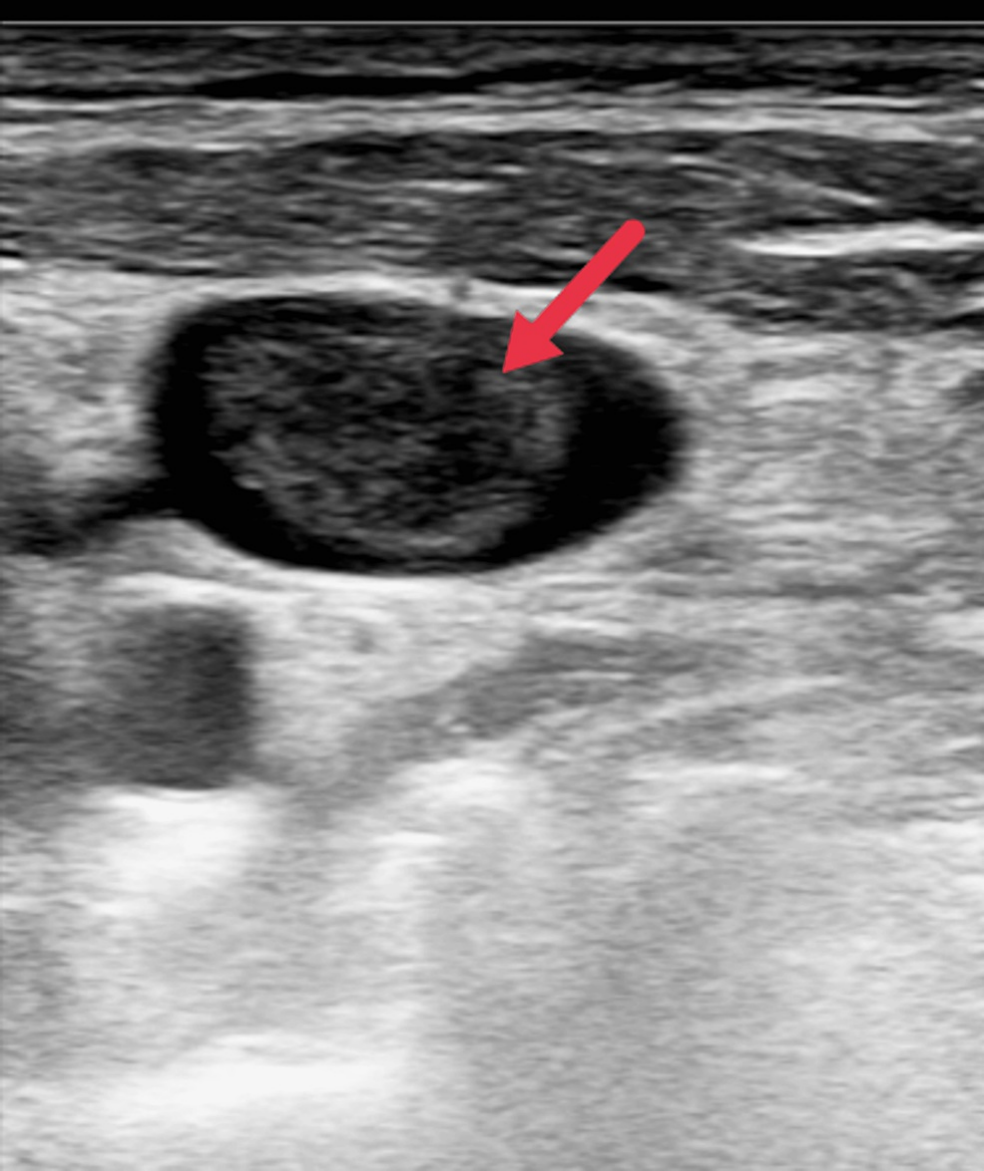
Point-of-care ultrasonography of left internal jugular vein showing echogenic thrombus (red arrow).

Ultrasonography of the abdomen and pelvis was done by a radiologist, which revealed the pancreatic head and body appearing bulky in size and mildly heterogenous, with peri-pancreatic fat stranding noted. A bilateral internal jugular vein (IJV) Doppler was also done, which showed an echogenic thrombus in the left IJV with no flow on the colour Doppler and was non-compressible, suggesting a thrombus in the left IJV (Figure 2).

**Figure 3:**
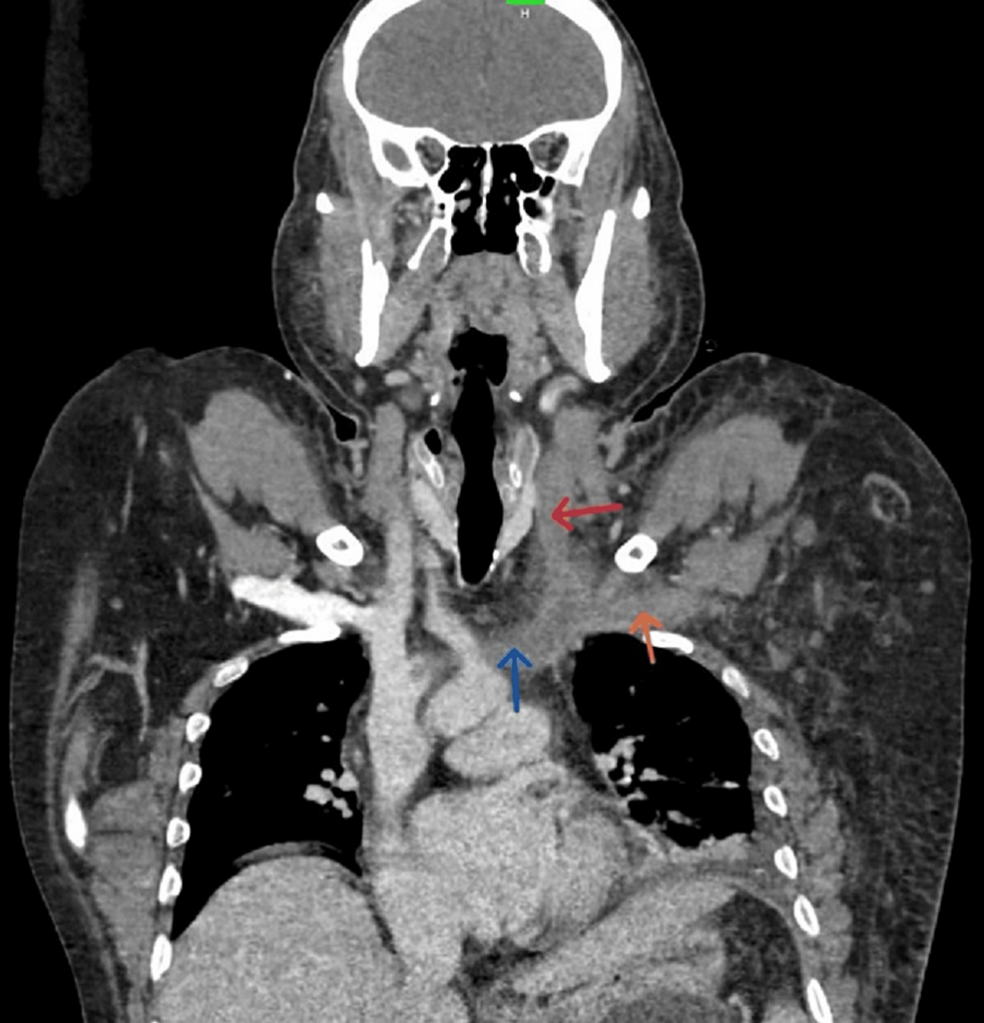
Coronal reformatted contrast-enhanced CT showing hypodense filling defects in left IJV (red arrow), left innominate vein (blue arrow), and left subclavian vein (orange arrow). Corresponding vessels on right show normal contrast opacification.

A contrast CT of the upper limb vessels and abdomen revealed near-complete thrombosis of the left ulnar veins, left radial veins, left brachial veins, left axillary vein, left subclavian vein, and superficial veins of the upper limb. The thrombus extended into the left IJV and left innominate vein (Figure 3).

**Figure 4:**
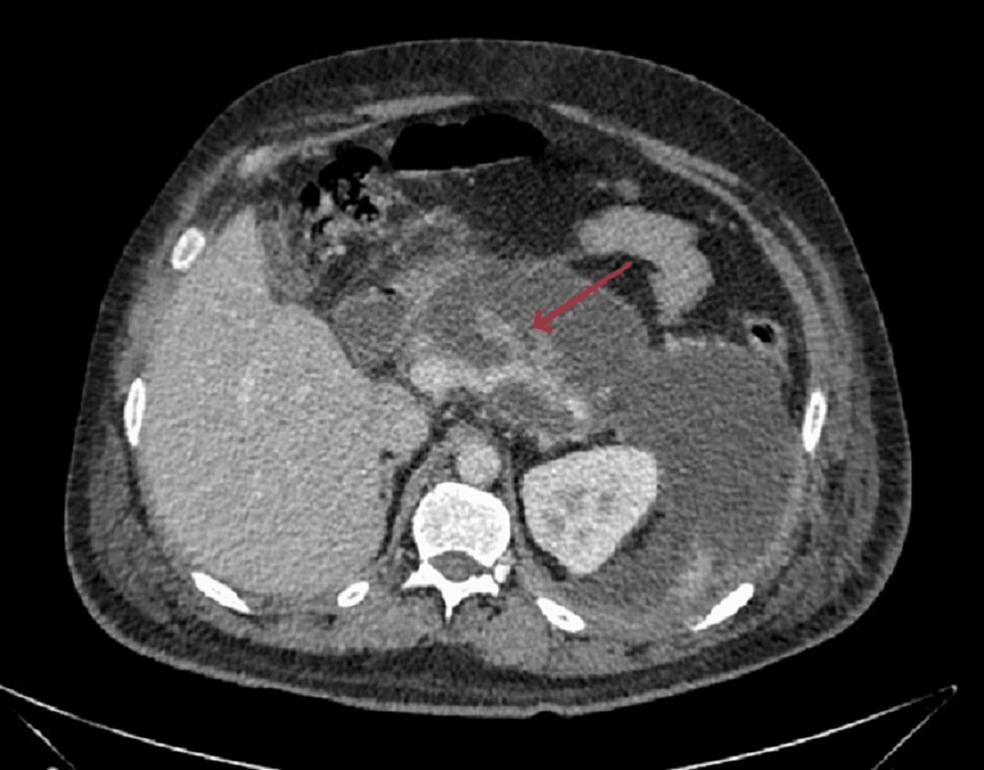
Axial contrast-enhanced CT showing multiple hypodense areas in the body region of pancreas (red arrow) - changes of necrotizing pancreatitis. Ill-defined diffuse fluid collection with enhancing wall in the anterior peripancreatic region extending along the greater curvature of the stomach, peripancreatic region.

Abdominal findings include multiple hypo-dense areas in the pancreas that were noted to be suggestive of necrotising pancreatitis, with an ill-defined diffuse fluid collection and an enhancing wall in the anterior peri-pancreatic region extending along the greater curvature of the stomach, peri-pancreatic region, into the left ilio-psoas, and also into the left inguinal region, likely suggestive of walled-off necrosis (Figure 4).

**Figure 5:**
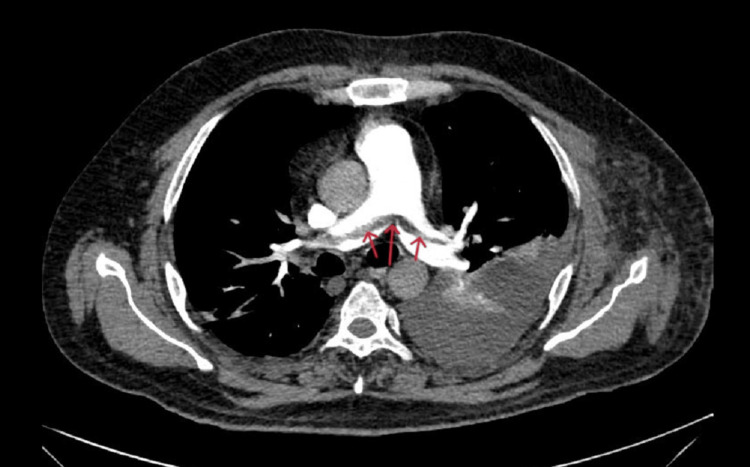
Axial section of CT pulmonary angiography showing hypodense filling defect (red arrows) at the bifurcation of the main pulmonary trunk extending to both right and left main pulmonary arteries.

CT pulmonary angiography was done to rule out pulmonary embolus, which revealed dilated main pulmonary trunk, right, and left pulmonary arteries with hypo-dense filling defects noted at bifurcation of the main pulmonary trunk extending into both the left and right pulmonary arteries (Figure 5).

The patient was started on conservative treatment for acute pancreatitis along with IV analgesics. The patient was started on anticoagulation for pulmonary thromboembolism and DVT and was later shifted to the ICU for further management. Despite thorough management, the patient succumbed to his illness after two days of an ICU stay.

## Discussion

The pancreas is a glandular organ located in the abdomen. It has both exocrine and endocrine functions. Exocrine function includes the secretion of pancreatic juice from acing cells into the duodenum through the pancreatic ducts [6]. The endocrine function of the pancreas is the release of hormones like insulin and glucagon from the pancreatic islets into the bloodstream. Acute pancreatitis is an inflammatory condition affecting the pancreas [6]. Patients with acute pancreatitis present to the emergency department with a history of severe epigastric pain. Causes of acute pancreatitis include alcohol, gallstones, drug-induced hyperlipidemia, post-endoscopic retrograde cholangiopancreatography (ERCP), autoimmune, and trauma, to name a few [6].

Diagnosing a case of acute pancreatitis is based on the revised Atlanta classification, which includes: epigastric pain associated with nausea and vomiting; serum lipase and amylase levels about 3 times the higher limit of the lab; and imaging of the abdomen with signs of pancreatitis [7]. The Atlanta classification categorises patients with acute pancreatitis if they meet two or three of the points listed in the criteria. The pancreatic juice contains digestive enzymes like trypsin in their inactive forms. Premature activation of trypsinogen in the pancreatic duct into trypsin leads to autodigestion of the gland, thereby causing acute pancreatitis [8]. The management of acute pancreatitis involves the use of aggressive IV fluids, with the choice of fluid being ringers lactate. Ringers Lactate must be started at 20 ml/kg bolus, followed by 3 ml/kg/hr [8].

The complications of acute pancreatitis are divided into local and systemic complications. According to the Atlanta classification, local complications include acute peripancreatic fluid collection, acute necrotic collection, walled-off necrosis, and pancreatic pseudocyst [9]. Systemic complications include systemic inflammatory response syndrome, disseminated intravascular coagulation, MODS, and death. Thrombosis is a well-reported complication associated with AP. Thrombosis is usually seen in severe pancreatitis, with acute necrotizing pancreatitis being more commonly associated with splanchnic venous thrombosis, leading to thrombosis of the splenic veins, superior mesenteric vein, or portal vein. It is theorised to be due to extrinsic compression of the splanchnic vessel system by an enlarged pancreas, causing stasis of blood [10].

Extra-splanchnic venous thrombosis is a rare complication that can be seen in acute pancreatitis. Pancreatic inflammation and the systemic inflammatory response play a vital role in the pathogenesis of extra-splanchnic thrombosis. Inflammation of the pancreas leads to the release of inflammatory modulators and cellular infiltration [11]. A hypercoagulable state is seen due to an increase in prothrombotic factors like fibrinogen that can stimulate the coagulation cascade, activate the platelets, and form fibrin-rich thrombin [12]. As a part of the host immune response, immune modulators like interleukins and cytokines get released from the inflamed pancreas. These can enter the systemic circulation, activating the coagulation pathways leading to the formation of thrombi in the systemic vasculature. Lastly, vascular changes due to proteolytic damage or inflammation may also play a significant part in splanchnic and extra-splanchnic venous thrombosis [13]. There is limited literature published regarding extra-splanchnic venous thrombosis as a complication of AP.

In a study done by Sissingh et al. [14], there is a consensus among pancreatologists to use therapeutic anticoagulation in the acute phase of AP, but there is a scarcity in the literature regarding the use of therapeutic anticoagulation in cases of extra-splanchnic venous thrombosis. To improve patient survival, the management of such a complication must incorporate consideration of the risk-benefit ratio before starting anticoagulation.

## Conclusions

Our case report highlights one of the rare complications seen in acute pancreatitis. Acute pancreatitis has high mortality and morbidity and needs prompt diagnosis and early treatment. Delay in treatment can lead to dreaded complications, including disseminated intravascular coagulation, multi-organ dysfunction syndrome, and death. Extra-splanchnic thrombosis is a rare complication seen in acute pancreatitis that can lead to pulmonary thromboembolism, which is a life-threatening complication. Familiarity with this complication and prompt treatment can reduce the mortality and morbidity associated with it.
